# Supplementary figures and images for: Domain specific phenotypic expansion associated with variants in MACF1
Source: medRxiv. 2025 Jun 28:2025.06.26.25330137. Preprint. [Version 1] doi: 10.1101/2025.06.26.25330137 (PMC12262753; doi:10.1101/2025.06.26.25330137)

Number of HPO Terms per Individual

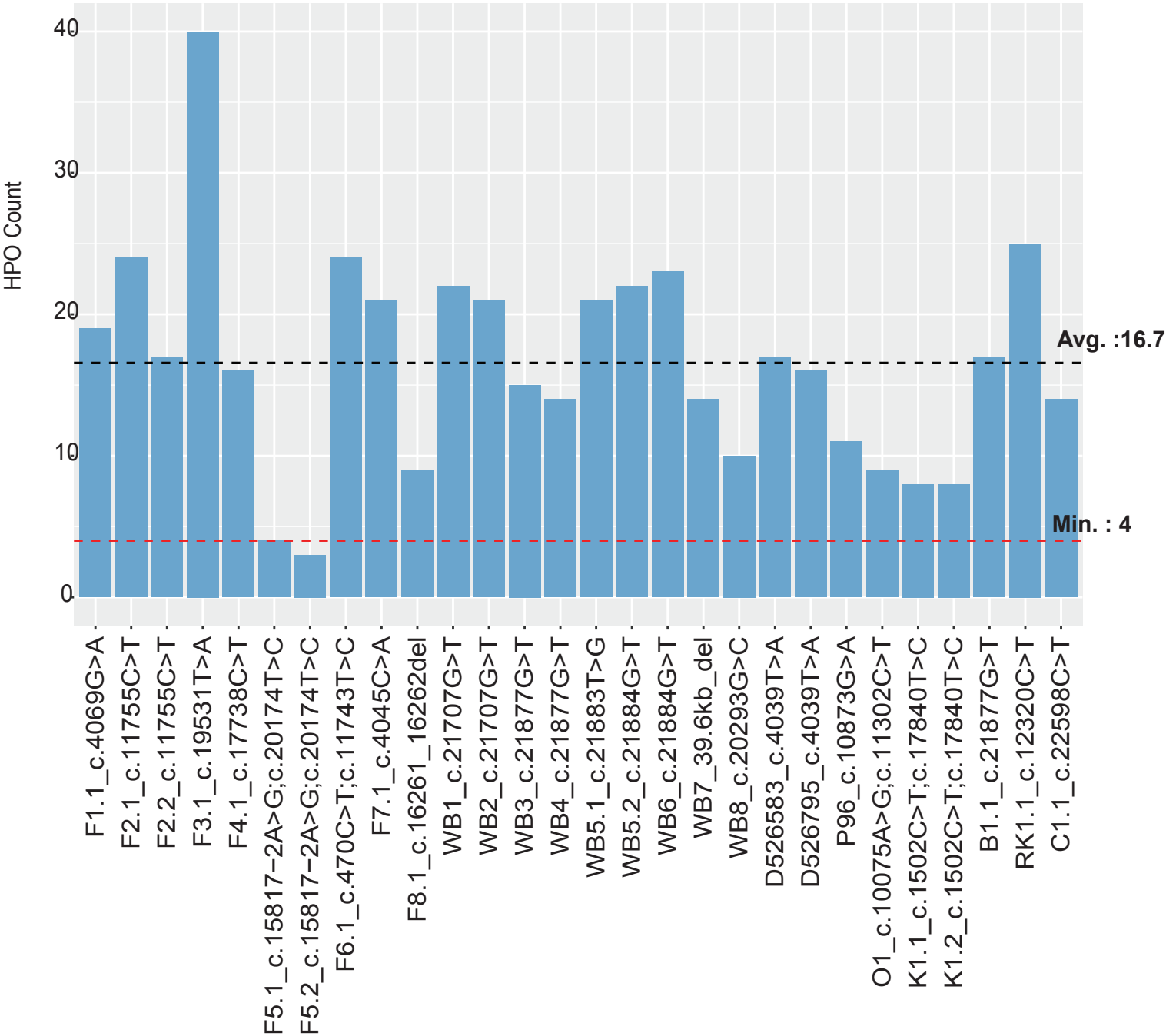

Supplement: Supplement 2 — Figure S1: Phenotypic depth. The number of HPO terms per individual is plotted. On average, there are 16.7 HPO terms per individual as represented by the black line. The red dotted line marks the minimum threshold of 4 HPO terms required for inclusion in the phenotypic clustering analysis. [file media-2.pdf]

**Individual 1**  
Chr1:39322647  
NM\_001394062.1:c.4069G>A, p.(E1357K)

19.9Mb

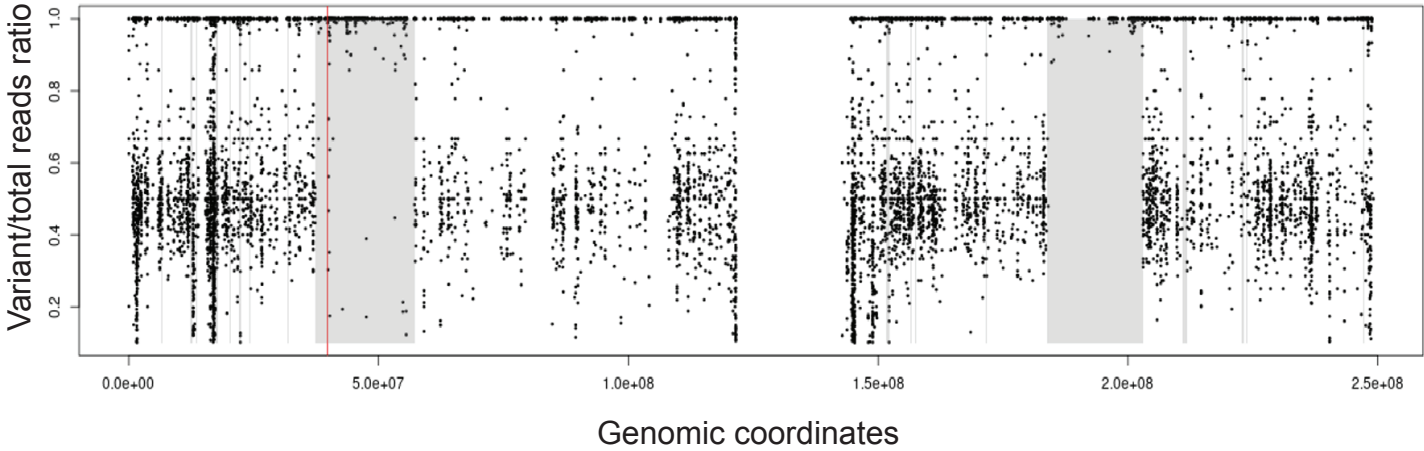

Supplement: Supplement 4 — Figure S3: The absence of heterozygosity (AOH) plot for Individual 1 demonstrates the variant (NM_001394062.1:c.4069G>A, p.(E1357K)) is located within a run of homozygosity (ROH) block of 19.9Mb on chromosome 1, marked by gray zones. [file media-4.pdf]
