# Supplementary material for: Domain specific phenotypic expansion associated with variants in MACF1
Source: medRxiv. 2025 Jun 28:2025.06.26.25330137. Preprint. [Version 1] doi: 10.1101/2025.06.26.25330137 (PMC12262753; doi:10.1101/2025.06.26.25330137)
Supplement: Supplement 3 — Figure S2: Gap Statistical Analysis for Determining Optimal Clusters. The gap statistic method compares the variation within observed data clusters against a null reference distribution to identify the optimal number of clusters with meaningful differences. (A) Gap statistic plot for all individuals in our cohort, where the largest gap difference indicates 2 optimal clusters. (B) Gap statistic plot for all individuals combined with OMIM gene term sets (p<0.001), identifying 2 optimal clusters. (C) Gap statistic plot for all individuals combined with OMIM disease term sets (p<0.001), identifying 4 optimal clusters. [file media-3.pdf]

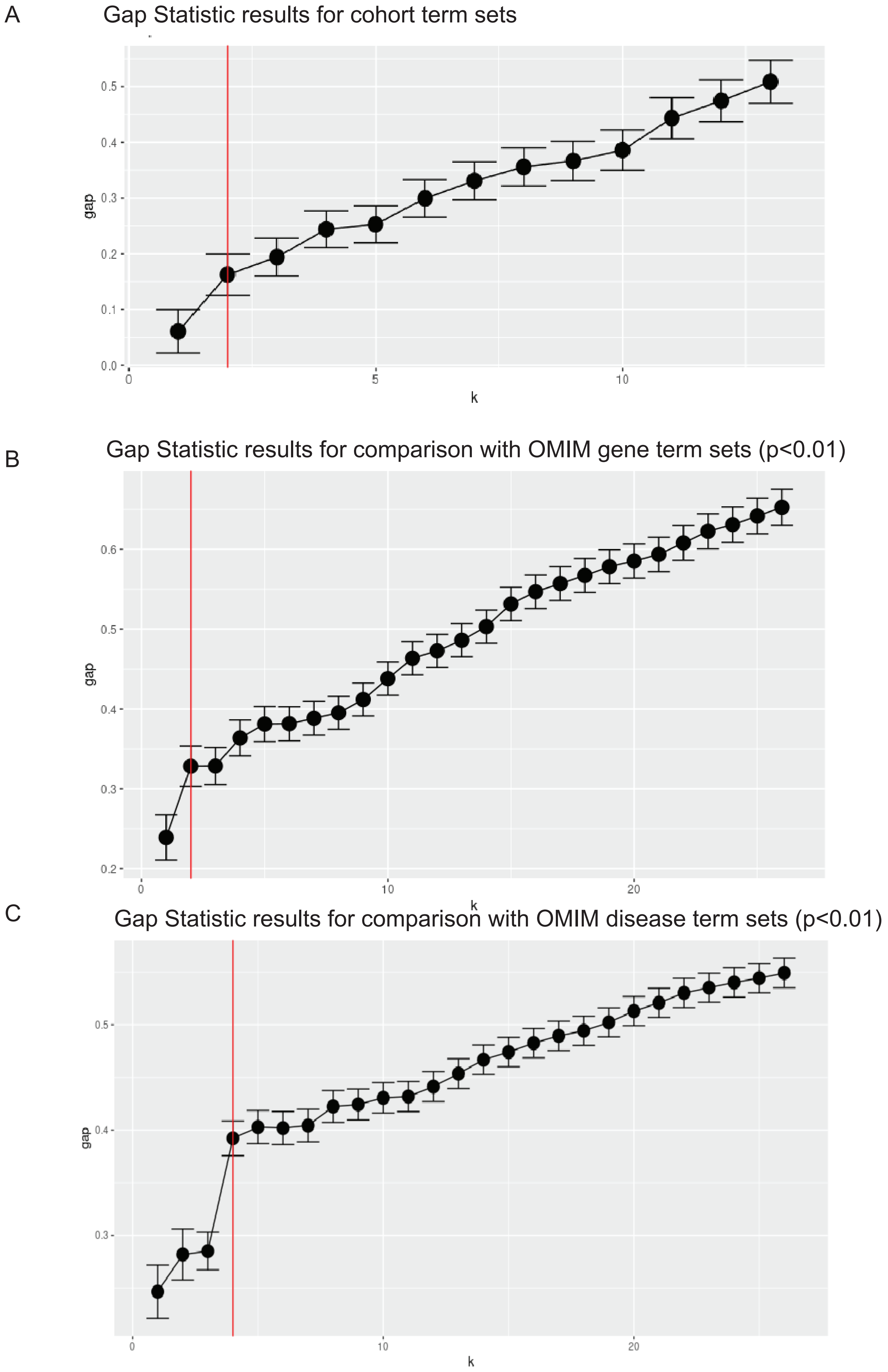
A
Gap Statistic results for cohort term sets
gap
0.0
0.1
0.2
0.3
0.4
0.5
0
5
10
k
B
Gap Statistic results for comparison with OMIM gene term sets (p<0.01)
gap
0.2
0.3
0.4
0.5
0.6
0
10
20
k
C
Gap Statistic results for comparison with OMIM disease term sets (p<0.01)
gap
0.3
0.4
0.5
0
10
20
k
